# Supplementary material for: Quantitative proteomics of infected macrophages reveals novel Leishmania virulence factors
Source: PLoS Pathog. 2026 Feb 10;22(2):e1013934. doi: 10.1371/journal.ppat.1013934 (PMC12931781; doi:10.1371/journal.ppat.1013934)
Supplement: S1 Fig — In parallel to the Leishmania spp.-infected BMDM proteome samples, Leishmania spp.-infected BMDM (MOI = 5) were stained with DiffQuick solutions and microscopically analysed to determine infection rates and the number of parasites per infected BMDM at different time points after addition of promastigotes. 8–10 visual fields with 60–120 BMDM each derived from 2 replicates were counted per group. Mean ± SD is shown. (PDF) [file ppat.1013934.s012.pdf]

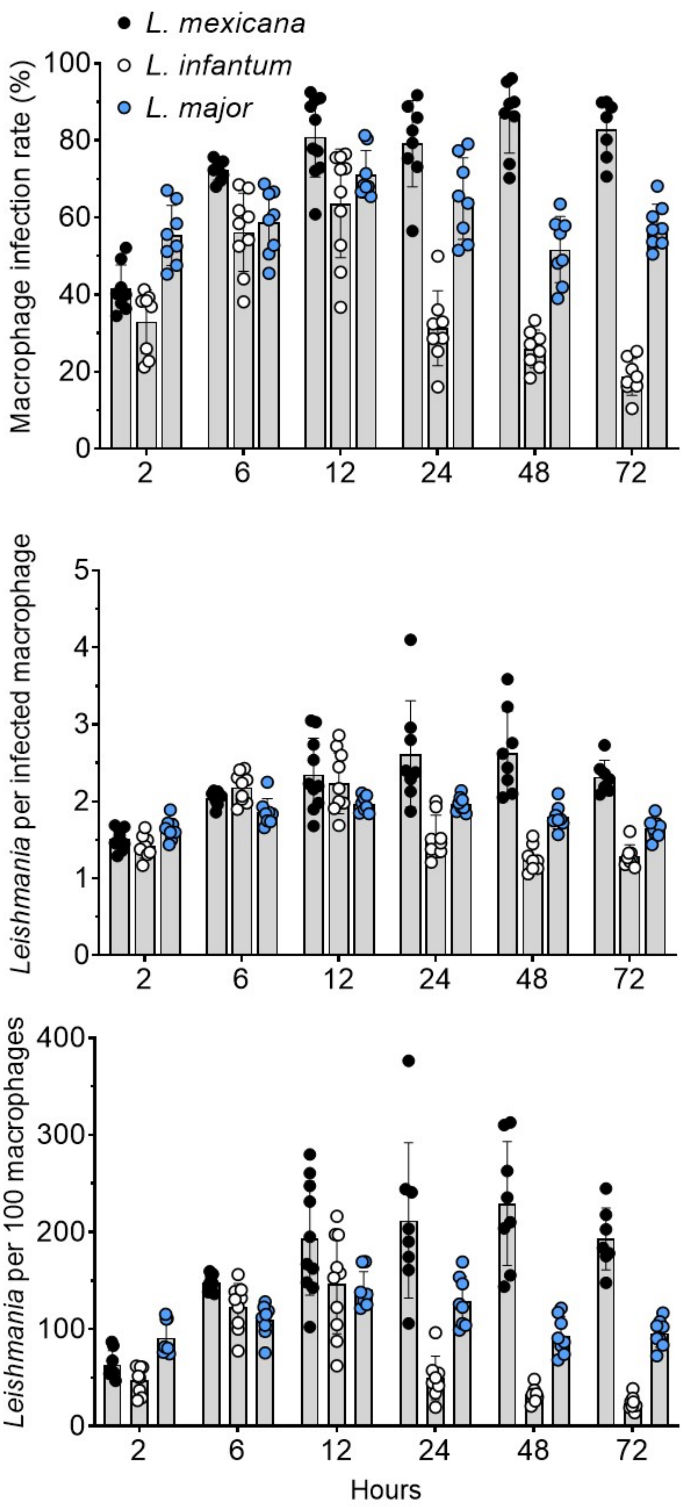

Supp. Fig. 1 Infection rate, number of amastigotes per infected macrophage and *Leishmania* per 100 macrophages at different time points of the culture. In parallel to the *Leishmania spp*.-infected BMDM proteome samples, *Leishmania spp*.-infected BMDM (MOI=5) were stained with DiffQuick solutions and microscopically analysed to determine infection rates and the number of parasites per infected BMDM at different time points after addition of promastigotes. 8-10 visual fields with 60-120 BMDM each derived from 2 replicates were counted per group. Mean ± SD is shown.
