# Supplementary material for: Quantitative proteomics of infected macrophages reveals novel Leishmania virulence factors
Source: PLoS Pathog. 2026 Feb 10;22(2):e1013934. doi: 10.1371/journal.ppat.1013934 (PMC12931781; doi:10.1371/journal.ppat.1013934)
Supplement: S2 Fig — a, Density plot showing the number of proteins IDs included per OrthoMCL ID for L. infantum (green), L. major (pink), and L. mexicana (blue). b, Barplot of the number of quantified OrthoMCL IDs per infection time course with the color indicating the number of quantified protein IDs associated to the quantified OrthoMCL ID. (PDF) [file ppat.1013934.s013.pdf]

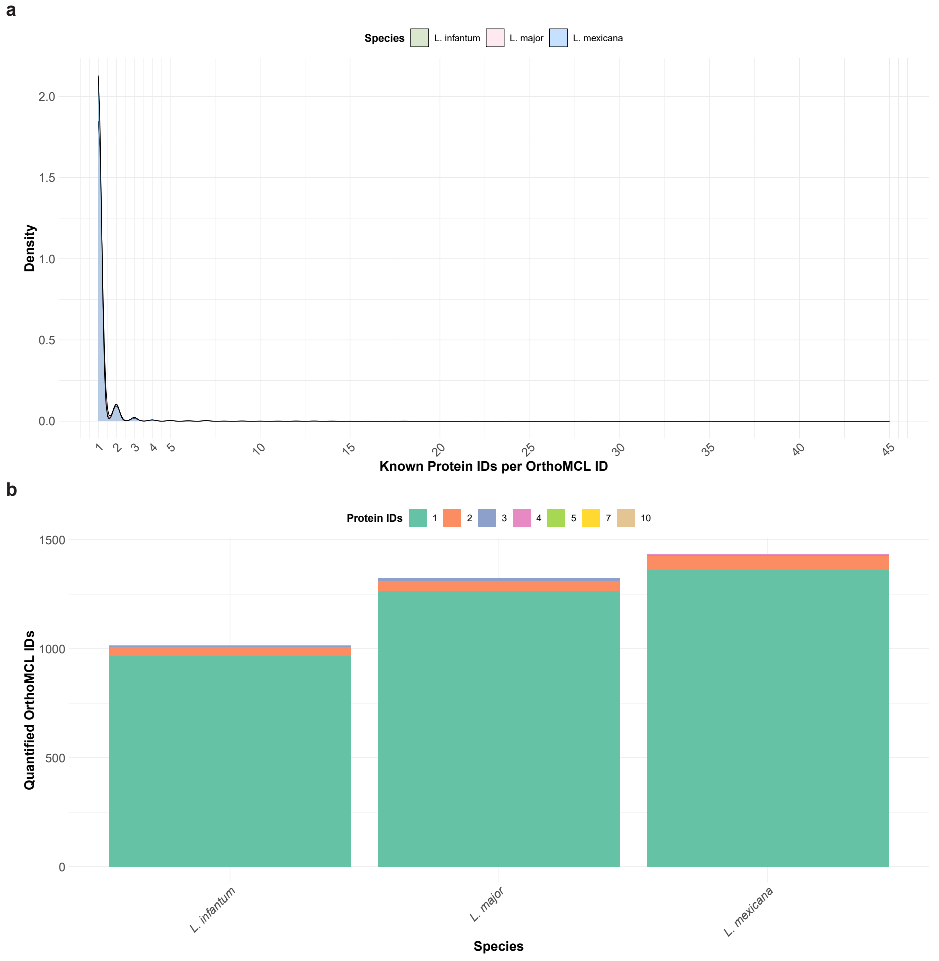

Supp. Fig. 2. ***Leishmania spp.*** **orhtologs. a,** Density plot showing the number of proteins IDs included per OrthoMCL ID for *L. infantum* (green), *L. major* (pink), and *L. mexicana* (blue). **b,** Barplot of the number of quantified OrthoMCL IDs per infection time course with the color indicating the number of quantified protein IDs associated to the quantified OrthoMCL ID
