# Supplementary material for: Quantitative proteomics of infected macrophages reveals novel Leishmania virulence factors
Source: PLoS Pathog. 2026 Feb 10;22(2):e1013934. doi: 10.1371/journal.ppat.1013934 (PMC12931781; doi:10.1371/journal.ppat.1013934)
Supplement: S3 Fig — a, Heatmap displaying the Spearman correlation coeficient for all measured samples in the L. infantum (green), L. major (pink) and L. mexicana (blue) infection time course experiments. Each row represents the correlation coeficient (light-to-dark blue sale) of each sample across the time course (grayscale). b, Violin plots displaying the distribution of the Euclidean distance between each sample from adjacent time points for the L. infantum (green), L. major (pink) and L. mexicana (blue) infection time course experiments. Differences between time courses are tested with Welch’s t-test (p-value < 0.05). (PDF) [file ppat.1013934.s014.pdf]

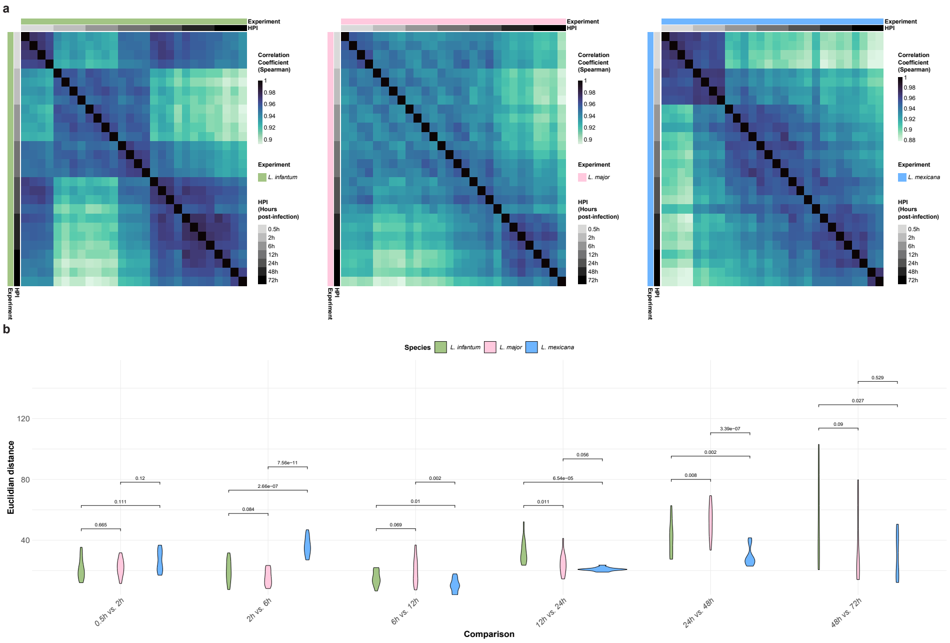

Supp. Fig. 3. Relation between proteome variancy and infection time course progression. a, Heatmap displaying the Spearman correlation coeficient for all measured samples in the *L. infantum* (green), *L. major* (pink) and *L. mexicana* (blue) infection time course experiments. Each row represents the correlation coeficient (light-to-dark blue sale) of each sample across the time course (grayscale). **b,** Violin plots displaying the distribution of the Euclidean distance between each sample from adjacent time points for the *L. infantum* (green), *L. major* (pink) and *L. mexicana* (blue) infection time course experiments. Differences between time courses are tested with Welch's t-t est (p-value < 0.05).
