# Supplementary material for: Quantitative proteomics of infected macrophages reveals novel Leishmania virulence factors
Source: PLoS Pathog. 2026 Feb 10;22(2):e1013934. doi: 10.1371/journal.ppat.1013934 (PMC12931781; doi:10.1371/journal.ppat.1013934)
Supplement: S4 Fig — Each row represents the abundance (z score [LFQ]; yellow-to-blue scale) of each quantified protein across the postinfection timepoints (grayscale). Proteins are annotated as either M. musculus (gray) or belonging to Leishmania spp. (green, pink, and blue for L. infantum, L. major, and L. mexicana, respectively). b, Line plot of highlighted proteins for the L. infantum (green), L. major (pink), and L. mexicana (blue) experiments. Each line represents the protein abundance (mean [log2{LFQ}]; y-axis) across the hours postinfection (x-axis). Lines are color coded using, pinkscale, or bluescale for L. infantum, L. major, and L. mexicana experiments, respectively. (PDF) [file ppat.1013934.s015.pdf]

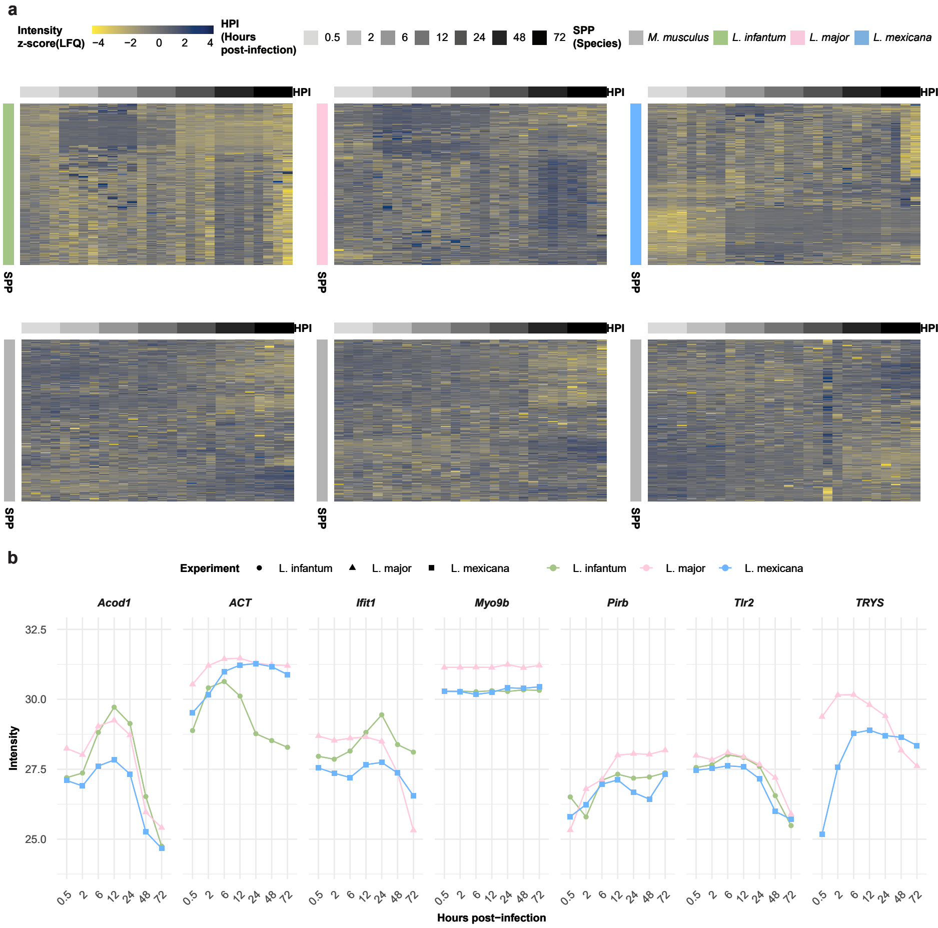

Supp. Fig. 4.***Protein expression profiles for the three Leishmania spp. experiments.* a,** Heatmap of quantified proteins for the L. infantum (green), L. major (pink), and L. mexicana (blue) experiments. Each row represents the abundance (z score [LFQ]; yellow-to-blue scale) of each quantified protein across the postinfection timepoints (grayscale). Proteins are annotated as either M. musculus (gray) or belonging to Leishmania spp. (green, pink, and blue for L. infantum, L. major, and L. mexicana, respectively). **b,** Line plot of highlighted proteins for the L. infantum (green), L. major (pink), and L. mexicana (blue) experiments. Each line represents the protein abundance (mean [log2{LFQ}]; y-axis) across the hours postinfection (x-axis). Lines are color coded using, pinkscale, or bluescale for L. infantum, L. major, and L. mexicana experiments, respectively.
