# Supplementary material for: Quantitative proteomics of infected macrophages reveals novel Leishmania virulence factors
Source: PLoS Pathog. 2026 Feb 10;22(2):e1013934. doi: 10.1371/journal.ppat.1013934 (PMC12931781; doi:10.1371/journal.ppat.1013934)
Supplement: S6 Fig — SOM analysis. a, Barplot showing the number quantified protein IDs before (0: Raw) and after filtering (1: Quantile filter, 2: IQR filter and 3: Orthology filter) prior to the SOM analysis for the L. infantum (green), L. major (pink) and L. mexicana (blue) infection time course experiments. b, SOM model. Each cell of the 2x3 hexagonal topology grid represents a cluster. Each panel shows, clock-wise, and starting at the upper-left panel: how cells are coloured depending on the overall distance to their nearest neighbors; a pie-chart depicting the representative vectors, where the radius of a wedge corresponds to its magnitude in a particular time point; how cells are coloured depending on the number of protein IDs; each protein ID depicted in its cell, based on how close they are to the representive vector. c, Boxplot showing the distribution of the distance between each protein ID and its winning unit. The mean is shown as a red dot, while the dashed line shows the distance’s 75th quantile. (PDF) [file ppat.1013934.s017.pdf]

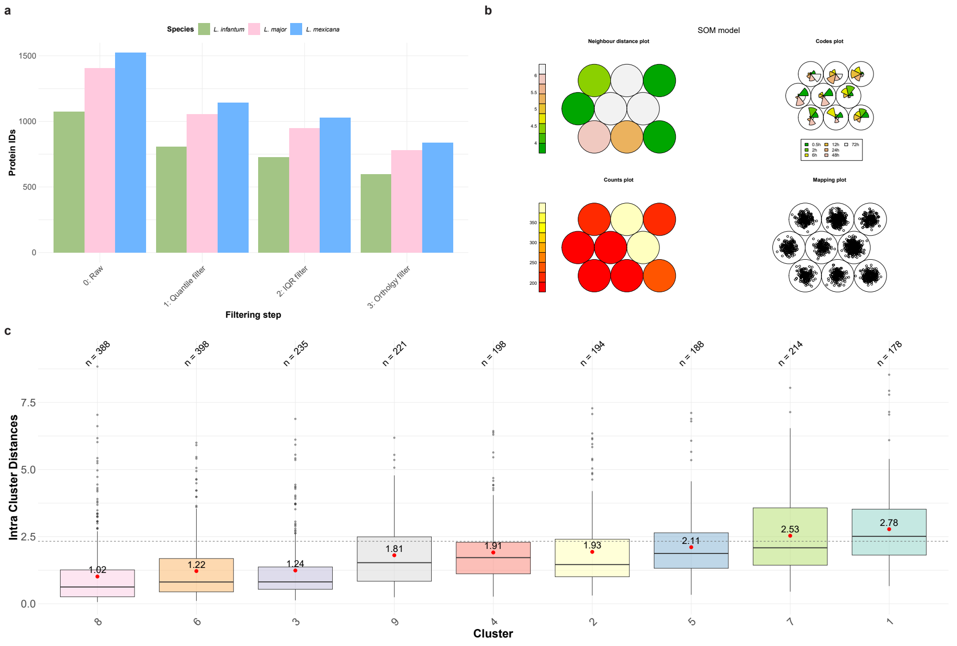

Supp. Fig. 6. *Leishmania Spp.* SOM analysis. a, Barplot showing the number quantified protein IDs before (0: Raw) and after filtering (1: Quantile filter, 2: IQR filter and 3: Orthology filter) prior to the SOM analysis for the *L. infantum* (green), *L. major* (pink) and *L. mexicana* (blue) infection time course experiments. **b,** SOM model. Each cell of the 2x3 hexagonal topology grid represents a cluster. Each panel shows, clock-wise, and starting at the upper-left panel: how cells are coloured depending on the overall distance to their nearest neighbors; a pie-chart depicting the representative vectors, where the radius of a wedge corresponds to its magnitude in a particular time point; how cells are coloured depending on the number of protein IDs; each protein ID depicted in its cell, based on how close they are to the representive vector. **c,** Boxplot showing the distribution of the distance between each protein ID and its winning unit. The mean is shown as a red dot, while the dashed line shows the distance's 75th quantile.
