# Supplementary material for: Quantitative proteomics of infected macrophages reveals novel Leishmania virulence factors
Source: PLoS Pathog. 2026 Feb 10;22(2):e1013934. doi: 10.1371/journal.ppat.1013934 (PMC12931781; doi:10.1371/journal.ppat.1013934)
Supplement: S8 Fig — a, Dot pot showing all significantly overrepresented (FDR < 0.05, blue-to-red scale) Hallmark signature terms (y-axis) and their gene ratio (x-axis). The dot size represents the number of included proteins from each particular term. (PDF) [file ppat.1013934.s019.pdf]

a

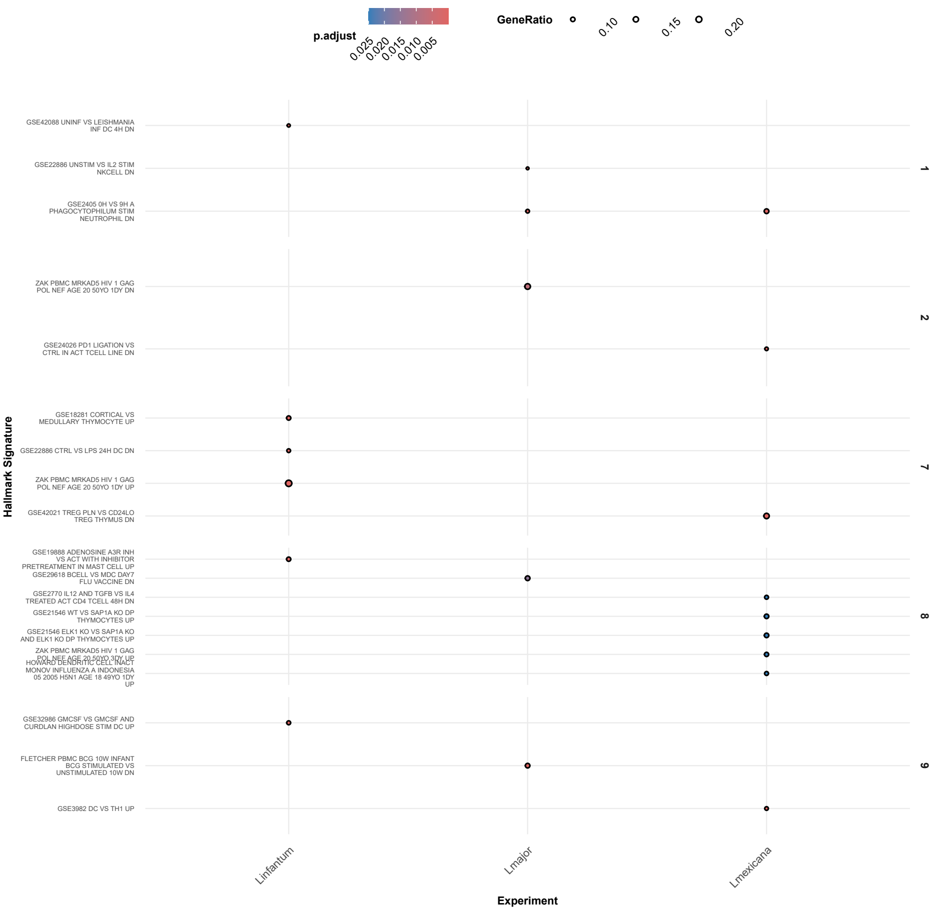

Supp. Fig. 8. ***M. musculus*** **Hallmark signature functional analysis. a,** Dot pot showing all significantly overrepresented (FDR < 0.05, blue-to-red scale) Hallmark signature terms (y-axis) and their gene ratio (x-axis). The dot size represents the number of included proteins from each particular term.
