# Supplementary figures and images for: Quantitative proteomics of infected macrophages reveals novel Leishmania virulence factors
Source: PLoS Pathog. 2026 Feb 10;22(2):e1013934. doi: 10.1371/journal.ppat.1013934 (PMC12931781; doi:10.1371/journal.ppat.1013934)

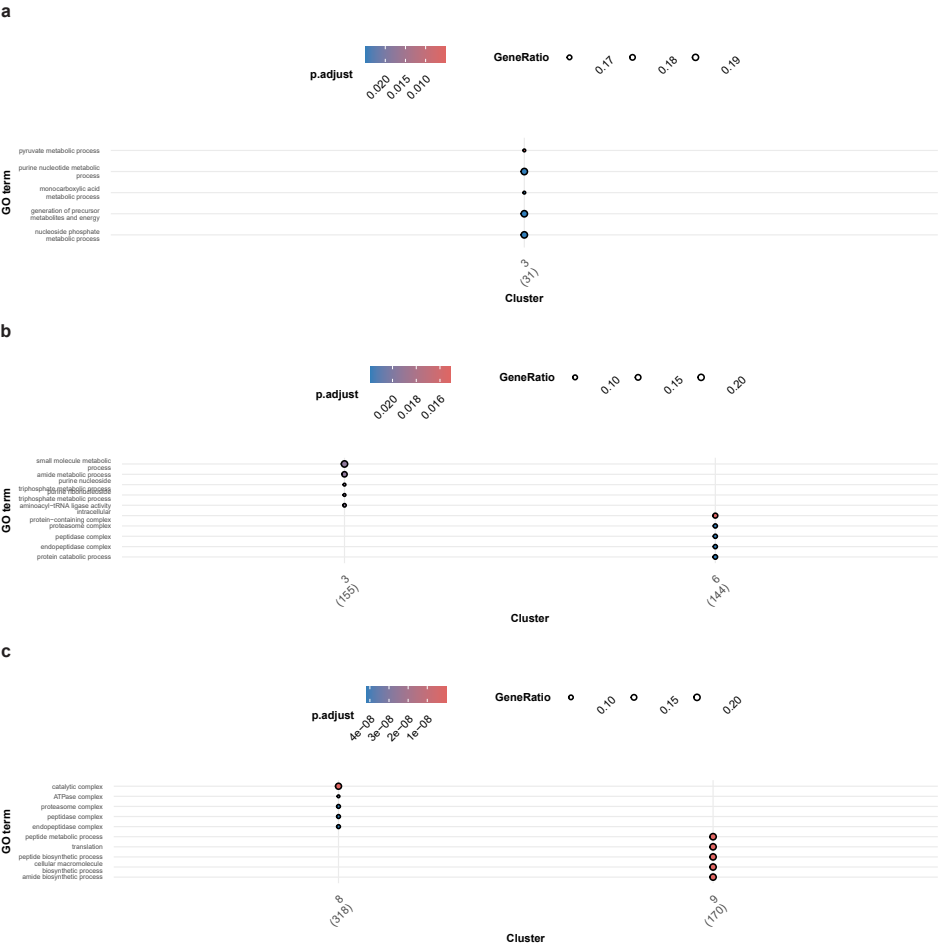

Supplement: S9 Fig — Dot pot showing all significantly overrepresented (FDR < 0.05,blue-to-red scale) GO BP signature terms (y-axis) and their gene ratio per cluster (x-axis) for the L. infantum (a), L. major (b) and L. mexicana (c) experiments. The dot size represents the number of included proteins from each particular term. (PDF) [file ppat.1013934.s020.pdf]
