# Supplementary material for: Quantitative proteomics of infected macrophages reveals novel Leishmania virulence factors
Source: PLoS Pathog. 2026 Feb 10;22(2):e1013934. doi: 10.1371/journal.ppat.1013934 (PMC12931781; doi:10.1371/journal.ppat.1013934)
Supplement: S10 Fig — Procyclic promastigote (left panels) and axenic amastigote (right panels) of ∆Lmx28.2260 (middle panels) and ∆Lmx10.0130 (bottom panels) populations show life cycle stage-specific morphologies, as observed by scanning electron microscopy. LmxCas9 cells (top panels) were analysed as a control. (PDF) [file ppat.1013934.s021.pdf]

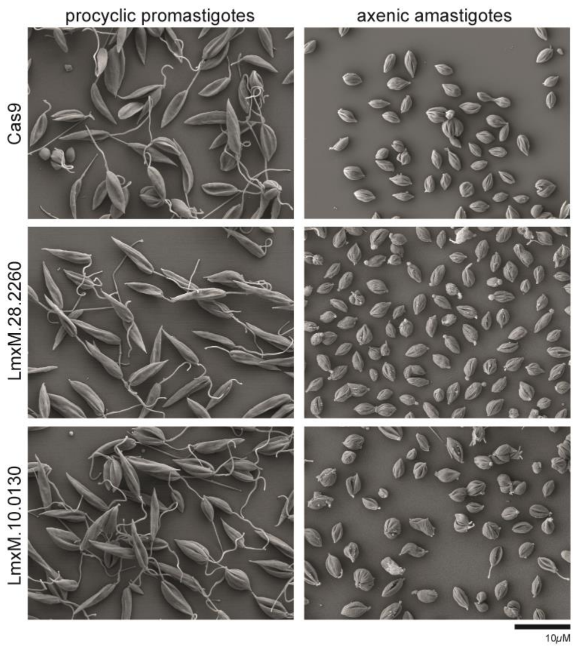

Supp. Fig. 10. *In vitro differentiation* of ∆Lmx28.2260 and ∆Lmx10.0130 . Procyclic promastigote (left panels) and axenic amastigote (right panels) of ∆Lmx28.2260 (middle panels) and ∆Lmx10.0130 (bottom panels) populations show life cycle stage-specific morphologies, as observed by scanning electron microscopy. LmxCas9 cells (top panels) were analysed as a control.
