# Supplementary material for: Quantitative proteomics of infected macrophages reveals novel Leishmania virulence factors
Source: PLoS Pathog. 2026 Feb 10;22(2):e1013934. doi: 10.1371/journal.ppat.1013934 (PMC12931781; doi:10.1371/journal.ppat.1013934)
Supplement: Attachment — Submitted filename: Response to the reviewers_20250903.docx [file ppat.1013934.s023.docx]

Point by point response to the reviewers

**Reviewers' Comments:**

Reviewer's Responses to Questions

**Part I - Summary**
Please use this section to discuss strengths/weaknesses of study, novelty/significance, general execution and scholarship.

**Reviewer #1:** The authors present a complex proteomic profiling of murine macrophages infected with three different species of Leishmania parasites at different time points. The species were chosen according to the clinical symptoms they cause: L. major causes cutaneous lesions, L. mexicana shows muco-cutaneous involvement and L. infantum presents a visceral tropism. The approach used is pertinent and generates valuable data regarding Leishmania and host cell responses during infection. However, important points are missing or not exploited in a comprehensive manner. In particular, the infection course of each parasite strain used in the macrophages is not described but necessary for the interpretation of the results (see major comments). The methods for data analyses and resulting plots are insufficiently described, and some choices are causing confusion, in particular analyzing host cell and Leishmania data together or separate depending on the figures. The impact of this manuscript could have been significantly increased by an in-depth systems analysis of the identified expression changes (e.g. GO enrichment, STRING network etc), which could render the rather descriptive data biologically more relevant. The discussion is a bit weak: the second paragraph is very repetitive, the third one pushes the idea of species-specific virulence factors, but without actual experimental proof, and the relevance of the approach used - in vitro infections of murine BMDMs - regarding in vivo infection is not discussed.

**Reviewer #2**: The authors of this paper carry out a comprehensive quantitative mass spectrometric analysis of the proteomes of three Leishmania species and Bone Marrow Derived Macrophages (BMDMs) over the course of an in vitro infection. They select a couple of putative L. mexicana virulence factors for further characterization as a proof of principle for functional significance during the infection. The work, along with publicly searchable database, contributes a valuable resource for the Leishmania, the ‘TriTryp’ community, and all those who study intracellular pathogens. The work is executed with rigor (experiments in quadruplicates) and I appreciated the interrogation of the early time points in the infection (0.5 h and 2 h). While the manuscript is generally written, I believe the authors can do a better job at integrating the results into existing knowledge and better highlighting the new insights gained into the infection dynamics of a Leishmania infection. The sections describing the parasite and macrophage protein expression results are a little anemic.

**Reviewer #3:** Thank you for the opportunity to review the manuscript entitled "Quantitative proteomics of infected macrophages reveals novel Leishmania virulence factors." Overall, the study presents interesting findings, and I appreciate the effort put into exploring the proteomics of Leishmania virulence factors. However, there are several key areas in the manuscript that require clarification and further development to enhance the overall impact of the work.

*These are all very helpful comments, and we appreciate the suggestions to improve the quality of our manuscript. In general, however, this study was designed to establish a resource platform that provides complementary data to ongoing studies in the community or starting points for new projects, similar to studies we have previously published in PLOS Pathogen and elsewhere (1, 2). We never intended to describe parasite cell biology processes mechanistically, as this would be an overinterpretation of our data. We agree with one of the reviewers that follow-up experimental validation is necessary to place our data into an infection biology context. While this is beyond the scope of this resource and surely projects of their own, we do currently investigate the function of several proteins in details that are unexpectedly involve in differentiation of Leishmania promastigotes to amastigotes.*

*To address the reviewers’ suggestions, we have added supplementary figures to better integrated our data into existing knowledge and revised the manuscript accordingly. We also provide the data that describes the infection dynamics of the different species used in this study. All changes are described in detail below.*

**Part II – Major Issues: Key Experiments Required for Acceptance**
Please use this section to detail the key new experiments or modifications of existing experiments that should be absolutely required to validate study conclusions.

Generally, there should be no more than 3 such required experiments or major modifications for a "Major Revision" recommendation. If more than 3 experiments are necessary to validate the study conclusions, then you are encouraged to recommend "Reject".

General comments:

**Reviewer #1:**
- A proper description of the course of each type of infection must be included. How does each parasite species perform in the macrophages over time, until the time point of 72H but also after? Are they cleared or do they proliferate? Is the dynamic similar for the three species or different? This is essential, as it could explain the different dynamics of expression profiles observed for each parasite species over time. The authors should not only show the number of infected cells (only metrics given in the Fig. 6) at each time point but also the number of parasites per 100 macrophages to better assess the quality of infection. These are key parameters used for in vitro macrophages infection studies.
*We agree that these data are necessary to better access the course of infection with the different parasites and provide them in a supplementary figure (Supp. Fig. 1).*

- The choice of performing the analyses on the host cell and Leishmania proteomes together or separately is not always pertinent in my opinion, and sometimes causes confusion. For example, I don’t see the point of doing the analyses presented in Figure 2 to pool both proteomes, as it renders the heatmaps useless. For the data shown in Figures 3 and 4: were the 9 clusters defined based on the total proteome (macrophage + Leishmania) and then only the mouse or parasite proteins were analyzed, or were the clusters defined on each proteome separately? How was the number of clusters (9 in each case) determined? This information should be clarified and included in the methods section.

*The mouse and Leishmania proteomic data are presented either together or separately depending on the specific analysis performed:*

*- For the exploratory data analysis (Figures 1C and 2A), which aimed to visualize the data set and summarize its main characteristics, we retained the structure of the raw data as much as possible. Since the proteomes of M. musculus and Leishmania spp. were measured simultaneously at each post-infection time point, a single combined dataset was obtained per experiment. Protein quantification steps (including normalization and missing value imputation) were performed on the full dataset, incorporating proteins from both host and parasite. Therefore, to reflect the context and dynamic range of protein abundance, we considered it more appropriate to display both species together in the same heatmap. That said, we acknowledge the value in separating host and parasite profiles to highlight species-specific trends, and have therefore included individual heatmaps for each species as a supplementary figure (Supp. Fig.3).*

*- For the self-organizing map (SOM) analysis (Figures 3 and 4), host and parasite datasets were analysed independently. The number of clusters (nine per species) was determined empirically, based on both intracluster distances (i.e., similarity of protein expression profiles within clusters) and neighbourhood distances between clusters to ensure meaningful separation (Supp. Figures 3B, 3C, 4B and 4C). Classical clustering evaluation methods, such as the elbow method, were tested but did not yield consistent or interpretable results for our data. A detailed description of the clustering approach and the underlying rationale has been added to the revised methods section.*

Detailed comments:
- Fig 1: Panel C: How were these PCA plots made? Was each infected time point compared to the non-infected control at time point 0, or to non-infected time-points at the same time in culture? This should be clearly explained in the text and methods. The proteome of (uninfected) macrophages in culture is likely to change over time, even when not infected. How was this aspect considered in the analyses?
*The PCA plots were generated using the base R prcomp function, which performs principal component analysis (PCA) on the proteomic data. PCA itself is a statistical technique that identifies patterns in data by transforming it into a set of linearly uncorrelated variables called principal components. To address the reviewer’s query about comparisons in PCA plots: PCA does not inherently compare specific time points directly. Instead, it visualizes the overall variance in the dataset, highlighting patterns and differences across samples. In our study, PCA was used to explore the overall variability and clustering of proteomic profiles across different experimental conditions, including infected and non-infected samples over time.*

*Regarding the variability of uninfected macrophages in culture over time, we acknowledge that such changes can occur. To mitigate this, experiments were conducted in quadruplicate to ensure robustness and consistency in our observations. This approach helps in discerning genuine biological differences from experimental variability. Furthermore, during the quantification of proteins using MaxQuant, a normalization step was employed to correct for potential technical variations and ensure reliable comparisons across samples.*

- I understand that these PCA plots combine the mouse and parasites proteins, but this should be clarified, as it is not obvious from the legend. It would be interesting to have separated PCA plots for only the mouse or the Leishmania proteins (maybe in a supplementary figure).

*We discussed this issue already in the general comment section above. Separate PCA plots can easily be accessed in our interactive database at* [*https://butterlab.imb-mainz.de/LInfDB/*](https://butterlab.imb-mainz.de/LInfDB/).

- Line 125: The authors write “The lower number of L. infantum proteins correlates with its lower invasion rates of macrophages, which resulted in a lower number of parasites in individual BMDMs”. Where is this very important information shown? Do you suggest that it is an explanation for the lower number of L. infantum proteins detected in your approach? *We changed the text to be more precise and provided the information in a supplementary figure (Supp. Fig. 1).*

- Fig 2: The choice of combining host cell and Leishmania proteomes eludes me. For panel A in particular, mixing the proteomes of the host cells and the pathogen renders the heatmap useless, as we can’t distinguish any pattern/clustering anymore.

*We discussed this issue in the general comment section above.*

Panel C, presenting the dynamic of expression of chosen factors over time, is interesting but needs to be put into perspective with the different dynamics of infection, that need to be added. If the result the authors want to illustrate is that some common proteins present different expression dynamics depending on the species, then they should present the dynamic of expression of these factors in the 3 species in the same graph (one graph per protein), at least in supplementary figures.

*We thank the reviewer for this suggestion and adapted the visualization accordingly: shared proteins are now shown in per-protein line plots across the three infection models in a supplementary figure (Supp. Fig. 4), allowing clearer comparison of their expression dynamics across species.*

- Fig 3-4: As mentioned above, it is not clear from the methods section if the SOM clusters were made from the total proteomes (host cell + Leishmania), or only on the host cell for Figure 3 and Leishmania for Figure 4. Given that these figures then show the expression profiles of host cell and pathogen proteomes separately, the SOM analysis should be done separately. Panels A and B should be inverted. As for Fig 2, the biological relevance of the data presented here is unclear. This needs to be rectified by (i) assessing the dynamics of infection of the macrophages with the 3 species, and (ii) including GO term enrichment analysis.
*Regarding the first point whether the SOM clusters were generated from the total proteomes (host cell + Leishmania) or separately: this has been addressed in the general comment section above. Concerning the suggestion to invert Panels A and B: our intention was to first present the total number of proteins per cluster (bar plots in Panel A) to give the reader an overview of the relative size and structure of the clusters. We then follow with Panel B, which shows the individual proteins in PCA space, allowing visualization of how well proteins are grouped within each cluster. We believe this order provides a logical flow from overview to detailed visualization. Regarding point (i): we now provide data describing the dynamics of infection in a supplementary figure (Supp. Fig. 1). Regarding point (ii): This was a very good suggestion. We have now addressed this by including additional analyses to enhance the biological interpretability of the data. Specifically, we have conducted Hallmark pathway enrichment analysis for the host (mouse) protein clusters and Gene Ontology (GO) enrichment analysis for the Leishmania clusters. These results have been included as supplementary figures 8 and 9, respectively.*

- Line 210: “Overall, we observed different temporal protein expression patterns for the Leishmania spp., with many virulence factors peaking earlier in L. infantum than in L. major and L. mexicana.” The authors need to show if this is due (or not) to differences in infection dynamics between the different parasite species.

*We now provide data describing the infection dynamics in a supplementary figure (Supp. Fig. 1). However, we only see significant differences at late time points.*

- Fig 5: Figure title and legend: “significant expression profile” (title) and “significant protein IDs”. What does ‘significant ‘ mean here? This should be reformulated if this does not refer to statistics. Panel B is not clear: what time points are considered here? The text only mentions “We overlapped all significantly up- and downregulated proteins”. When?

*The term "significant" refers to proteins that, in any of the pairwise time point comparisons included in the plot, which meet the following criteria: a p-value ≤ 0.05 (Welch’s t test) and an absolute fold change ≥ 2. This is detailed in the Methods section, under the "Bioinformatics Analysis" subsection, in the paragraph describing the pairwise analysis. Regarding Panel B, all time points were considered. The overlap shown includes all protein IDs that were found to be significant in at least one of the pairwise comparisons, regardless of the specific time point at which the significance was observed.*

- Fig 6: A method section to describe the process of differentiation from promastigotes to amastigotes in culture and the readout used for successful differentiation (cumulative cell density) is needed. In panels D and H: the percentage of infected cells is not enough to properly follow the infections, as some infected cells could contain very few parasites and other hundreds. The authors need to include the number of parasites per 100 macrophages to assess the distribution of parasites. Panel F: if the dynamic of expression of the protein PFR-2 is used as a read-out for amastigote differentiation in culture, then it should be presented at the beginning of the figure, in a separated panel and for all 3 Leishmania species. Microscopy pictures to illustrate the differentiation of amastigotes (or amastigote-like forms) in culture would be appreciated in Sup. Figures (line 303-306: “The knockout cells were unable to proliferate as axenic amastigotes, although they had the morphological characteristics of amastigotes, such as a rounded cell body and a shortened flagellum”).

*We added the differentiation protocol to the material & methods section. Panel D and H: we provided data describing the infection rates and dynamics of macrophage infections with L. mexicana in a supplementary figure (Supp. Fig. 1). We never observed changes of infection rates or parasites per infected macrophage during the entire course of infection.* *Panel F: As light microscopy is not suitable to monitor changes in morphology during the differentiation process, we provided scanning EM images of differentiated parasite in a supplementary figure as requested (Supp. Fig. 10).*

- Discussion: line 352-355: “Experiments with this protein also underscore the differences in the infection dynamics among the three species, as this protein was significantly regulated only in L. mexicana but not in L. infantum or L. major”. Here you imply that this protein has no role in the virulence of L. major or L. infantum, but the only way to know it is a loss-of-function study in L. major and L. infantum. Idem at 366: “Given the differences in the protein dynamics of LmxM.28.2260 and its orthologs in L. major and L. infantum, it is possible that the protein plays species-specific roles in the infection process”. Either show experimental evidence for this or strongly tone down.

*We changed the text and mentioned that further experiments are necessary.*

**Reviewer #2:**

Fig. 2 panel -This panel is not pretty but informativ

*We are happy that the reviewer appreciates the content of this panel.*

Fig. 3 – I am not sure I agree with the interpretation of the self-organizing map analysis and with the assertion that that ‘infection response of the host cell to different parasite species is generally similar’. One sees similar trends (as expected you lump the three datasets in the same clustering analysis), but most protein IDs are not shared. The more striking observation is that the proteins underlying those trends are mostly specific to the infections by different species of parasites. The response seems to be quite heterogeneous and infection species-specific! Only 383 mouse proteins behave identically across all 9 clusters. An attempt to understand/dissect the apparent species-specific response would be important here.

*We agree with your assessment and have accordingly toned down our emphasis on the similarities between the host responses to different Leishmania species in the headline. To better address the species-specific aspects of the host and parasite responses, we have now conducted additional functional analyses for both the mouse and Leishmania clusters. These analyses aim to dissect and highlight the specific biological processes and pathways associated with each cluster, providing a more detailed understanding of the species-specific responses.*

ln 194 – In the same vein, the statement “ there is a large overlap in M. musculus proteome expression changes, with a similar temporal pattern” reflects an anticipated result given the nature of the analysis which is aimed at doing exactly that.

*We discussed this point above in the general comments section of reviewer #1.*

Fig. 3A – I appreciate what the authors are trying to represent in Fig. 3A. but the bar plot (stacked or not) is not the most accurate given the redundant proteins identified in the response to species-specific infections. I believe the Venn diagrams I panel C are sufficient.
The same comment applies to Fig. 4A (despite the fact that the overlaps are less significant here)
*The bar plots in Fig. 3A and Fig. 4A provide a general overview of the total number of proteins per cluster before focusing on the overlaps shown in the Venn diagrams. We believe that presenting the total distribution first helps to contextualize the extent and structure of each cluster, making it easier for the reader to interpret the overlaps and species-specific differences shown subsequently.*

Ln 210-212 ‘Overall, we observed different temporal protein expression patterns for the Leishmania spp., with many virulence factors peaking earlier…’. This statement is made as a summary for a section where no reference to virulence factors is made. What is the definition of virulence factors in this paper? And consequently, what criteria were used for selecting the proof of principle proteins? Such filters, and the resulting subsets, would be of interest to the readers.

*Since a precise definition of the term virulence factor is still a topic of discussion in the field, we decided to use the term in a broader sense, which includes all factors that contribute to the ability of the parasite to cause the disease by enhancing infectivity, survival, or damage-causing potential in the host. This includes proteins that are needed for efficient stage differentiation, because this process is crucial to establish an infection in macrophages. However, we changed the text because we do not have an experimental proof that all of these proteins are indeed virulence factors.*

*There were no specific criteria for selection of the proof-of-principle proteins. They were randomly pick from the cohort of proteins with unknown function that are up-regulated during the cause of infection.*

**Reviewer #3:**

INTRODUCTION
• Line 48: The statement made requires citation, as there is still ongoing debate regarding the relationship between species and pathology in Leishmania. A reference would strengthen this assertion.

*We added a recent review.*

• Line 60: The statement is misleading as it implies that only Lutzomyia and Phebotomus species are involved in transmission. Please revise to reflect the involvement of other genera in Leishmania transmission.

*We changed the manuscript accordingly.*

• Line 80: While I understand that this study focuses on L. major, L. mexicana, and L. infantum, the manuscript would benefit from a broader discussion of other epidemiologically relevant species. Briefly contextualizing this research within a broader species framework would provide greater relevance to the field.

*We agree with the reviewer that the manuscript would benefit of a broader discussion of other leishmania species. Unfortunately, this is not possible (as mentioned above) because we reached the words restrictions for the main text already and need to be as concise as possible.*

• Line 90: The hypothesis for using only these three species is not clearly defined. It would be helpful to explain the rationale for selecting these species in particular. The readers should also gain better insight into the utility of quantitative proteomics in the context of Leishmania research, as this is not well conveyed in the introduction.
*The selection of these species is a trade-off among several aspects that we aimed to acknowledge. (i) Since the study was designed as a resource for the community, we wanted to use Leishmania strains that are broadly used for research. (ii) We wanted to include “old world” and “new world” strains. (iii) We wanted to include species causing cutaneous and visceral Leihmaniasis (A limitation here was that we were not allowed to work with L. donovani when we started the project). (iv) We needed to include our laboratory strain (L. mexicana) for follow-up experiments. (v) We wanted to take total world-wide infections and clinical impact into account.*

METHODS

• Line 384: Species names should be italicized for consistency with scientific writing standards. Also, the WHO nomenclature for the strains used is missing and should be provided for clarity.

*We provided this information.*

• Line 404: The rationale behind the selection of the multiplicity of infection (MOI) is not sufficiently explained. Was the same MOI used for all three species? If so, why? The assumption of no differences in species infectivity is not supported, and this needs to be addressed more explicitly.

*We obtain optimal infection rates with MOI 5 with all species. The data are now provided in a supplementary figure (Fig. Sup. 1).*

• Line 409: The methodology section lacks clarity regarding the timing of proteome extraction. It would be helpful to include information on the time points of infection used, the number of replicates, and how infection was quantified and selected. Providing infection plots would be beneficial here.

*We included this in Fig.1 and the first paragraph of the results section. A supplementary figure with data about the individual infections was provided (Sup. Fig 1).*

• Line 470: The rationale for transfecting L. mexicana is not well-justified at this point in the manuscript. While this is clarified in the results, it would be helpful for the reader to understand the reason behind this decision earlier in the methods section.

*The initial infections for the mass spec. analysis were done in the Schleicher lab, which works routinely with several species. The follow-up studies were done in the Janzen lab. Cell culture, genetic manipulation and infection experiments were only established for L. mexicana in this laboratory.*

RESULTS
• The results are intriguing, particularly the identification of shortlisted virulence factors. However, validation of these findings using additional methods is crucial. The data would benefit from further validation via independent approaches, especially considering the power of the proteomics data at hand.

• Given the comprehensive nature of the proteomics data, I would expect a more thorough exploration of the protein interactome. The inclusion of detailed analyses using well-established tools, such as AlphaFold Multimer, HADDOCK, ClusPro, and PyMOL for visualization, would elevate the findings significantly. Furthermore, using RVFScan for virulence factor gene detection and incorporating BioNumerics or Python for data analysis could provide additional insights into protein interactions and host-pathogen dynamics.

*While we agree that exploring the protein interactome would provide valuable insights, we would like to clarify that such conclusions cannot be derived directly from proteomic expression data alone. Determining specific protein–protein interactions requires dedicated experimental approaches, such as co-immunoprecipitation followed by mass spectrometry, cross-linking assays, or proximity labeling techniques. These methods were beyond the scope of the current study. We therefore chose not to apply in silico interaction prediction tools, as their results would lack experimental validation in this context.*

DISCUSSION
• The discussion section feels underdeveloped and overly speculative. While the authors provide some interpretation of the results, I would appreciate a deeper analysis of how these findings might translate from mice models to human infection. Additionally, the authors should be more transparent in discussing the limitations of their study. Given the scope of the research, there are several limitations that are not adequately addressed.

*How this finding might translate from mice to humans might also be quite speculative. To this end, a new study with human BMDMs might be more informative in the future.*

**Part III – Minor Issues: Editorial and Data Presentation Modifications**
Please use this section for editorial suggestions as well as relatively minor modifications of existing data that would enhance clarity.

**Reviewer #1:**

- Line 33: “…demonstrating the utility of our dataset”: this formulation is unclear and should be rephrased.

*We rephrased the sentence to make it clearer.*

- Line 64-66: “Once inside the host cell, the parasites interfere with the phagocytic pathway, resulting in a delay in phagolysosome maturation and, thus, elimination of the parasites”: the sentence is unclear, rephrase.

*We rephrased the sentence accordingly.*

- Line 94-96: “Only one study has investigated the dynamic changes that occur during the process of infection with L. amazonensis and L. major parasites in human macrophages, via transcriptomics”: I would reformulate to “only one study has used transcriptomics to investigate…”

*We changed the manuscript accordingly.*

- Line 100: “murine BMDMs”

*We changed the manuscript accordingly.*

- Line 132: “The lower number of quantified proteins among the three species also coincided with a lower number of species-exclusive proteins”: this sentence is unclear, rephrase.

*We rephrased the sentence.*

- Line 152: “Gini score” (not Gine)

*We changed the manuscript accordingly.*

- Line 251: “We tagged the gene in a cell line expressing GFP-GAPDH to investigate the localization of our candidate in glycosomal compartments of L. mexicana. Our microscopy images revealed that the LmxM.28.2260 signal clearly overlapped with that of GAPDH”. This formulation is unclear, as we don’t understand that GAPDH is used as a glycosomal marker.

*In trypanosomatids (including Leishmania) glyceraldehyde-3-phosphate dehydrogenase is exclusively localized to glycosomes and extracellular vesicles and is commonly used as a glycosomal marker for imaging analysis.*

- Line 257: “altered” should be replaced by modified or changed

*We changed the manuscript accordingly.*

- Line 295: “Although L. infantum and L. major orthologs were also quantified, we only measured a significant difference between time points in L. mexicana”. This is unclear: you didn’t detect the protein in the other species, or its expression wasn’t changing over time?

*We changed the manuscript accordingly.*

- Line 327-342: the paragraph is looping, repeating the same idea in 3 different formulations (the pathology is driven by the parasite not the host). Also, it should be discussed in this paragraph that this is an in vitro infection system and its shortcoming. How relevant are your data for an in vivo setting?

*We changed the manuscript accordingly.*

- Line 409: Protein extracts (not proteome)

*We changed the manuscript accordingly.*

- Line 453: “For protein dynamicity analysis, protein abundance stability across embryonic developmental stages was evaluated via the Gini coefficient”: embryonic developmental stages?? I guess this comes from a previous publication. To be removed.

*We changed the manuscript accordingly.*

**Reviewer #2:**

In the discussion of Fig.2 results, abundance and intensity are used interchangeably. Perhaps stick to one?

*Yes, we will stick to abundance and changed the text accordingly.*

**Reviewer #3:**

See above

1.	Dejung M, Subota I, Bucerius F, Dindar G, Freiwald A, Engstler M, et al. Quantitative Proteomics Uncovers Novel Factors Involved in Developmental Differentiation of *Trypanosoma brucei.* PLoS Pathog. 2016;12(2):e1005439.

2.	Butter F, Bucerius F, Michel M, Cicova Z, Mann M, Janzen CJ. Comparative proteomics of two life cycle stages of stable isotope-labeled *Trypanosoma brucei* reveals novel components of the parasite's host adaptation machinery. Mol Cell Proteomics. 2013;12(1):172-9.
