# Supplementary material for: Quantitative proteomics of infected macrophages reveals novel Leishmania virulence factors
Source: PLoS Pathog. 2026 Feb 10;22(2):e1013934. doi: 10.1371/journal.ppat.1013934 (PMC12931781; doi:10.1371/journal.ppat.1013934)
Supplement: Attachment — Submitted filename: Response to the reviewers 2026.docx [file ppat.1013934.s024.docx]

Point by point response to the reviewers

Dear Editor

Thank you for your letter and the reviewer's comments on our revised manuscript. We were happy to learn that the reviewer's comments on our manuscript were positive. We have addressed all the comments and suggestions below in details. We believe this manuscript improved due to the reviewer’s suggestions and hope that you consider the revised manuscript for publication in *PLOS Pathogens.*

Sincerely,

Christian J. Janzen and Falk Butter

**Additional Editor Comments:**

Please make the minor revisions requested by Reviewer 1.

*We modified supplementary figure S1 as requested by reviewer #1 and edited the manuscript.*

**Reviewers' Comments:**

**Part I - Summary**

Reviewer #1: This study presents a complex proteomic profiling of murine BMDMs infected with three different species of Leishmania parasites, chosen because they induce different clinical manifestations, at different time points. The work presented is well executed and provides a valuable resource for researchers investigating Leishmania parasites. In the revised version of their manuscript, the authors added important informations and analyses (in particular a better description of the dynamic of infection of each parasite species in the BMDMs) and modified the text accordingly. I do appreciate that some previous statements were cleraly toned down, to avoid over-interpretation of data.The resulting manuscript is more accurate and to my opinion only requires minor modifications.

Reviewer #4: (No Response)

**Part II – Major Issues: Key Experiments Required for Acceptance**

Reviewer #1: (No Response)

Reviewer #4: (No Response)

**Part III – Minor Issues: Editorial and Data Presentation Modifications**

Reviewer #1: - I thank the authors for adding the Supp. Figure 1, showing the dynamic of infection of each parasite species in BMDMs other time, answering my biggest concern. These results actually show that infantum tends to be cleared other time in this cells, contrary to mexicana who seems to establish a productive infection and proliferate, and could actually explain, at least in part, the dynamic of expression of some proteins (there is just less parasites for infantum, not really less protein per parasite). This has been sort of noted in the manuscript, but could be better integrated in relation to some results (in particular Fig 6). Of note, a graph showing the number of parasites per 100 macrophages (which can be deduced from the percentage of infected cells and the mean number of parasites per infected cell) would be appreciated (and was asked previously as a major comment).

*We edited Supp. Figure 1 and added the number of parasites per 100 macrophages as requested.*

- In relation to that point, I suggest to change this sentence to reflect this result: line 294: “This finding was consistent with our previous observation of species-specific protein expression dynamics during the time course of infection (Fig.4) and also correlates with the dynamic of infection profiles of the 3 parasites (Supp. Fig1).

*We changed the manuscript accordingly.*

- As it is written currently in the method section, the SOM analysis is still not super clear to me: I suggest that the authors simply include the answer they gave to the reviewing inside the method section: “For the self-organizing map (SOM) analysis (Figures 3 and 4), host and parasite datasets were analysed independently. The number of clusters (nine per species) was determined empirically, based on both intracluster distances (i.e., similarity of protein expression profiles within clusters) and neighbourhood distances between clusters to ensure meaningful separation”

*We added this sentence to the material and methods section.*

- Concerning the Fig 5 title and legend: “significant expression profile” (title) and “significant protein IDs”. I thank the authors for their explanation of what is significant in their study, but the point I was making here was purely semantic: there is no such thing as a “significant ID” or “significant profile”. I saw that this was modified in the text, but please modify the title of the figure (I suggest “Pairwise differential… with significantly modulated expression profiles”

*We apologise for the misunderstanding. We changed the title of Fig. 5 as suggested.*

- For the GFP-GAPDH sentence: even if I agree with the authors that GAPDH is a classical marker of glycosomes in trypanosomatids, it will not be obvious to every reader, especially those not familiar with the model. I think you just need a clear sentence in that part of the text saying that GFP-GAPDH is used as a marker of the glycosomal compartment. Your sentence “We tagged the gene in a cell line expressing GFP-GAPDH to investigate the localization of our candidate in glycosomal compartments of L. mexicana.” is ambiguous.

*We agree with the reviewer and revised the sentence for clarity.*

Reviewer #4: (No Response)
